# Deep learning enables edge deployment for citrus leaf disease recognition in natural orchard scenes

**DOI:** 10.3389/fpls.2026.1896487

**Published:** 2026-08-03

**Authors:** Zhaobo Huang, Shitong Fan, Chao Ma, Yanchao Yang, Peixuan Li, Wenfeng Li, Huan Zou

**Affiliations:** Yunnan International Joint Laboratory of Smart Crop Production, Yunnan Agricultural University, Kunming, China

**Keywords:** agricultural machine vision, citrus leaf disease recognition, deep learning, edge deployment, intelligent phytoprotection, lightweight object detection, natural orchard scenes, SKM-YOLOv11

## Abstract

**Background:**

Accurate and efficient detection of citrus leaf diseases is important for orchard monitoring, early intervention, and intelligent disease management. However, practical application in natural orchard environments remains challenging because of complex backgrounds, large variation in symptom scale, strong interclass similarity, and limited edge computing resources.

**Methods:**

In this study, a lightweight object detection model, SKM-YOLOv11, was developed based on YOLOv11n for citrus leaf disease recognition in natural scenes. A self-built dataset was constructed from public images and field images, containing 6414 images across six classes. StarNet-S050 was introduced as the backbone, C3k2-Star was designed to enhance feature fusion across scales, and a lightweight shared detection head, MNS-Head, was constructed to reduce prediction redundancy.

**Results:**

Compared with YOLOv11n, SKM-YOLOv11 increased Precision, Recall, and mAP @0.5 by 2.92, 1.47, and 0.95 percentage points, respectively. Meanwhile, FLOPs, parameter count, and model size were reduced by 31.7%, 32.7%, and 34.55%, respectively. Edge deployment on Jetson Orin NX Super achieved an inference speed of 72.45 frames per second.

**Conclusion:**

The proposed model has strong potential for real-time citrus disease screening on resource-constrained devices and provides a feasible solution for edge-based intelligent disease management in natural orchards.

## 1 Introduction

Accurate recognition of citrus leaf diseases is directly related to orchard disease monitoring, control decision making, and stable yield and quality improvement. With the development of smart agriculture and precision phytoprotection, intelligent disease diagnosis based on machine vision and deep learning has become an important technical pathway for orchard information perception. Compared with traditional manual inspection, deep learning methods have clear advantages in recognition efficiency, objectivity, and scalable application. However, in real production scenarios, recognition accuracy, robustness, and deployment efficiency must still be considered simultaneously (Ferentinos, 2018; Dananjayan et al., 2022; Zhang et al., 2022; Khan et al., 2022).

Research on visual recognition of citrus leaf diseases has gradually shifted from traditional methods based on handcrafted color, texture, and morphological features to deep learning-based segmentation, detection, disease classification, lightweight inference, and edge deployment in natural scenes (Mzoughi and Yahiaoui, 2023). Dananjayan et al. (2022) systematically evaluated multiple citrus leaf disease detection models in terms of recognition accuracy, inference speed, and resource consumption, demonstrating that this task requires not only high detection accuracy but also computational efficiency. Zhang et al. (2022) and Zhang et al. (2024a) further investigated citrus disease recognition and detection in complex orchard environments, showing that natural backgrounds, leaf occlusion, and variation in lesion scale can substantially affect model performance. In recent years, lightweight disease detection models have been widely applied to different crops. For example, studies on tomato, vegetable, cauliflower, rice, and peanut disease recognition have shown that YOLO series models have good speed advantages and deployment potential in agricultural vision detection (Qi et al., 2022; Li et al., 2022; Uddin et al., 2024; Pan et al., 2025; Sun et al., 2025; Zhang et al., 2024b). In citrus disease scenarios, Mo and Wei (2024) and Feng et al. (2025) also demonstrated that lightweight structures and attention mechanisms can improve detection performance in complex agricultural environments.

Although these studies provide an important foundation for visual recognition of citrus diseases, existing methods still require further improvement. First, some studies are based primarily on relatively regular or single background datasets for model design and validation. Integrated modeling of occlusion, background clutter, and uneven illumination in natural orchards remains insufficient, leading to limited stability in complex environments (Dananjayan et al., 2022; Zhang et al., 2022; Hu et al., 2023; Mo and Wei, 2024; Feng et al., 2025). Second, most existing improvements focus on single module optimization, such as enhancing only backbone feature extraction, introducing only an attention mechanism, or modifying only the detection head (Wang et al., 2023). The coupling among fine grained lesion feature extraction, semantic fusion across scales, and lightweight prediction has not been fully addressed (Wang and Liu, 2024; Miao et al., 2025; Qin et al., 2025; Bao et al., 2025; Li et al., 2025). In addition, edge deployment has become an important direction in agricultural vision research. However, many current methods still primarily target accuracy improvement, with insufficient systematic consideration of the balance among parameter count, computational cost, model size, and real time performance (Lin et al., 2024; Yang et al., 2025; Zhang et al., 2025). Therefore, achieving high accuracy citrus leaf disease recognition and efficient deployment in complex natural scenes remains a key research problem.

In this study, YOLOv11n was used as the baseline framework to develop a lightweight detection model suitable for complex natural scenes and targeting typical citrus leaf diseases, including citrus black spot, citrus greening, citrus canker, greasy spot, and algal spot. This study focused on three problems. First, under conditions where complex backgrounds and fine grained symptoms coexist, the network needs to enhance its representation of disease textures, lesion edges, and weak feature regions. Second, when lesions of multiple scales are present simultaneously, effective fusion between shallow spatial details and deep semantic information needs to be improved. Third, while maintaining high detection accuracy, model complexity must be further compressed to support real time deployment on edge devices. These problems directly correspond to the main bottlenecks in current citrus leaf disease detection in terms of accuracy, robustness, and deployability (Dananjayan et al., 2022; Hu et al., 2023; Mo and Wei, 2024; Feng et al., 2025; Zhang et al., 2024a).

To address these problems, this study proposed SKM-YOLOv11, a lightweight citrus leaf disease detection model with coordinated improvements to the backbone, feature fusion module, and detection head. Unlike previous lightweight citrus disease detectors that generally optimize feature extraction, attention, feature fusion, or prediction efficiency as relatively independent components, SKM-YOLOv11 treats backbone compression, multi scale feature adaptation, and shared prediction as a coupled design process. StarNet-S050 was introduced into the backbone to reduce redundant computation while retaining effective nonlinear feature interaction for subtle disease textures. C3k2-Star was constructed in the neck to recalibrate channel responses, align multi scale semantics, and adapt the compact backbone features before shared prediction. MNS-Head was then designed to reuse prediction parameters across P3, P4, and P5, thereby reducing redundancy and computational cost in the detection branches. This sequential pathway addresses the chained problems of insufficient basic representation, interscale feature inconsistency after backbone compression, and excessive detection head cost, thereby balancing detection performance and model efficiency.

The main methodological contribution of this study lies not in the isolated use of lightweight operators, but in the coordinated architecture that explicitly addresses representation compatibility between a compact backbone and a shared detection head. This design relationship was further examined through module combination ablation experiments, which clarified the adaptation role of C3k2-Star between StarNet-S050 and MNS-Head. From an application perspective, the proposed model supports automatic disease inspection, assisted diagnosis, and real time edge recognition in smart orchards. The experimental results showed that SKM-YOLOv11 reduced model complexity while improving detection accuracy and achieved real time inference on the Jetson Orin NX platform, confirming its practical potential for edge based citrus disease screening.

## 2 Materials and methods

### 2.1 Dataset construction

**Figure 1:**
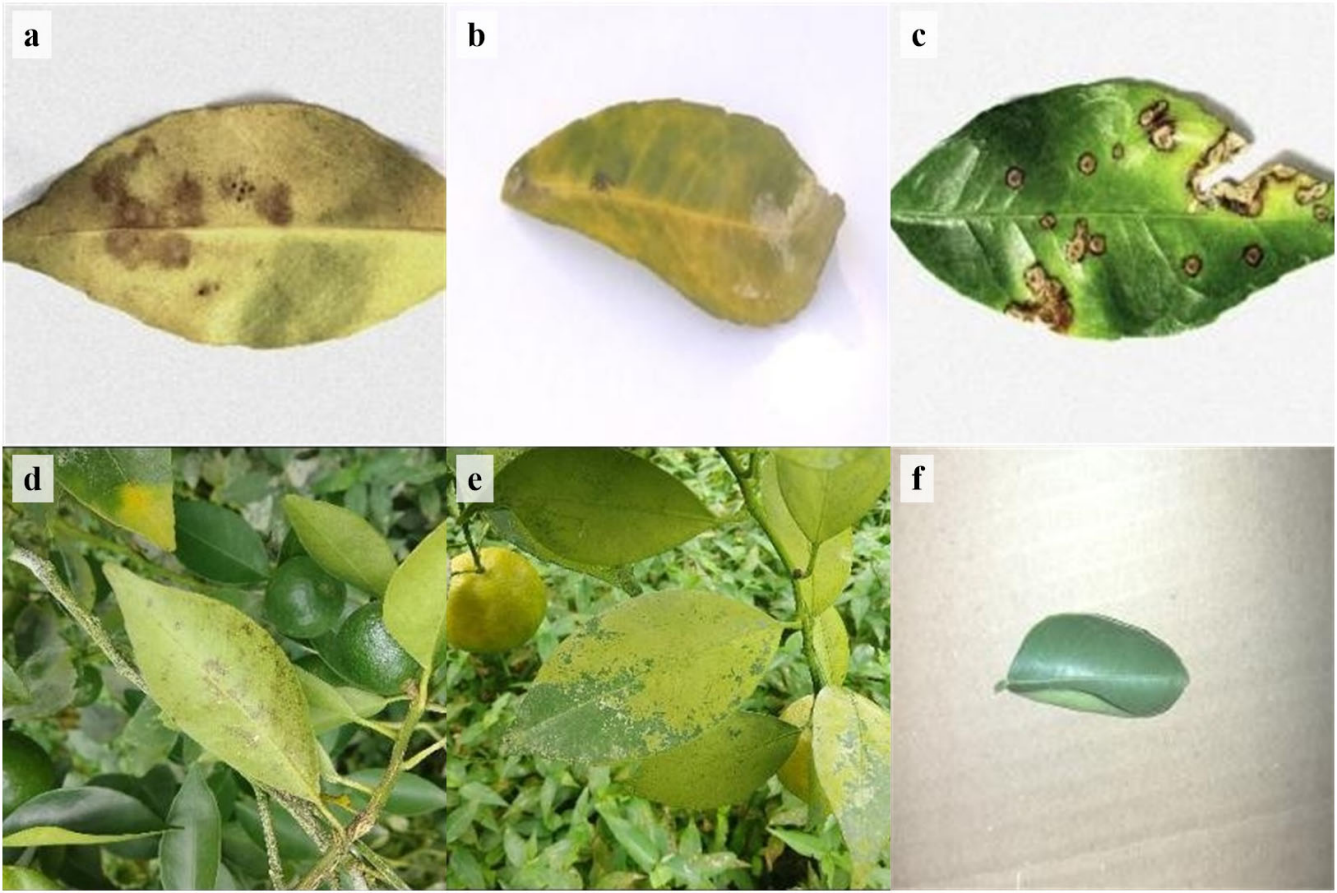
Examples of samples from the citrus leaf disease detection dataset. **(a)** Black spot. **(b)** Citrus greening. **(c)** Citrus canker. **(d)** Greasy spot. **(e)** Algal spot. **(f)** Healthy.

The citrus leaf disease detection dataset used in this study consisted of two parts. One part was obtained from the public AI Studio platform, including citrus black spot (259 images), citrus canker (591 images), citrus greening (360 images), and healthy leaves (357 images). The other part was collected from orchards and included images of greasy spot (257 images) and algal spot (257 images). Field images were collected under natural illumination using a mobile imaging device and included variations in leaf pose, illumination, background interference, and symptom appearance. The original dataset contained 2076 images. Representative samples are shown in **Figure 1**.

The LabelImg annotation tool was used to annotate target bounding boxes. In natural orchard scenes, citrus leaves are frequently distributed within overlapping canopies and dense foliage, where occlusion, background clutter, and uneven illumination make the boundaries of individual lesions difficult to distinguish. Many disease symptoms also appear as multiple small and spatially scattered lesions on the same leaf. Annotating each lesion separately would therefore produce numerous small or overlapping bounding boxes, increase annotation ambiguity, and introduce additional prediction and postprocessing costs during edge inference. For these reasons, the whole leaf region was selected as the annotation target rather than individual lesion regions. This strategy preserved the overall leaf contour, vein structure, texture characteristics, and spatial distribution of disease symptoms while limiting the number of detection targets. The task in this study was consequently defined as leaf level disease screening rather than lesion level localization or severity quantification. The annotation results were saved in TXT format.

Some images contained multiple leaves belonging to different disease categories. Each visible leaf was annotated using an independent bounding box, and each bounding box was assigned a single class label according to the symptoms of the corresponding leaf. Therefore, the presence of multiple disease categories referred to different labeled leaves within the same image rather than multiple class labels assigned to the same bounding box. During training, each ground truth bounding box was treated as an independent detection target. For each matched target, the classification loss was calculated using its corresponding single class label, while the bounding box regression loss and distribution focal loss were calculated using the coordinates of the associated ground truth box. When bounding boxes partially overlapped because of leaf occlusion, they retained independent coordinates and class labels and were processed as separate targets. Therefore, overlapping leaves did not introduce conflicting classification labels or duplicated regression supervision.

**Figure 2:**
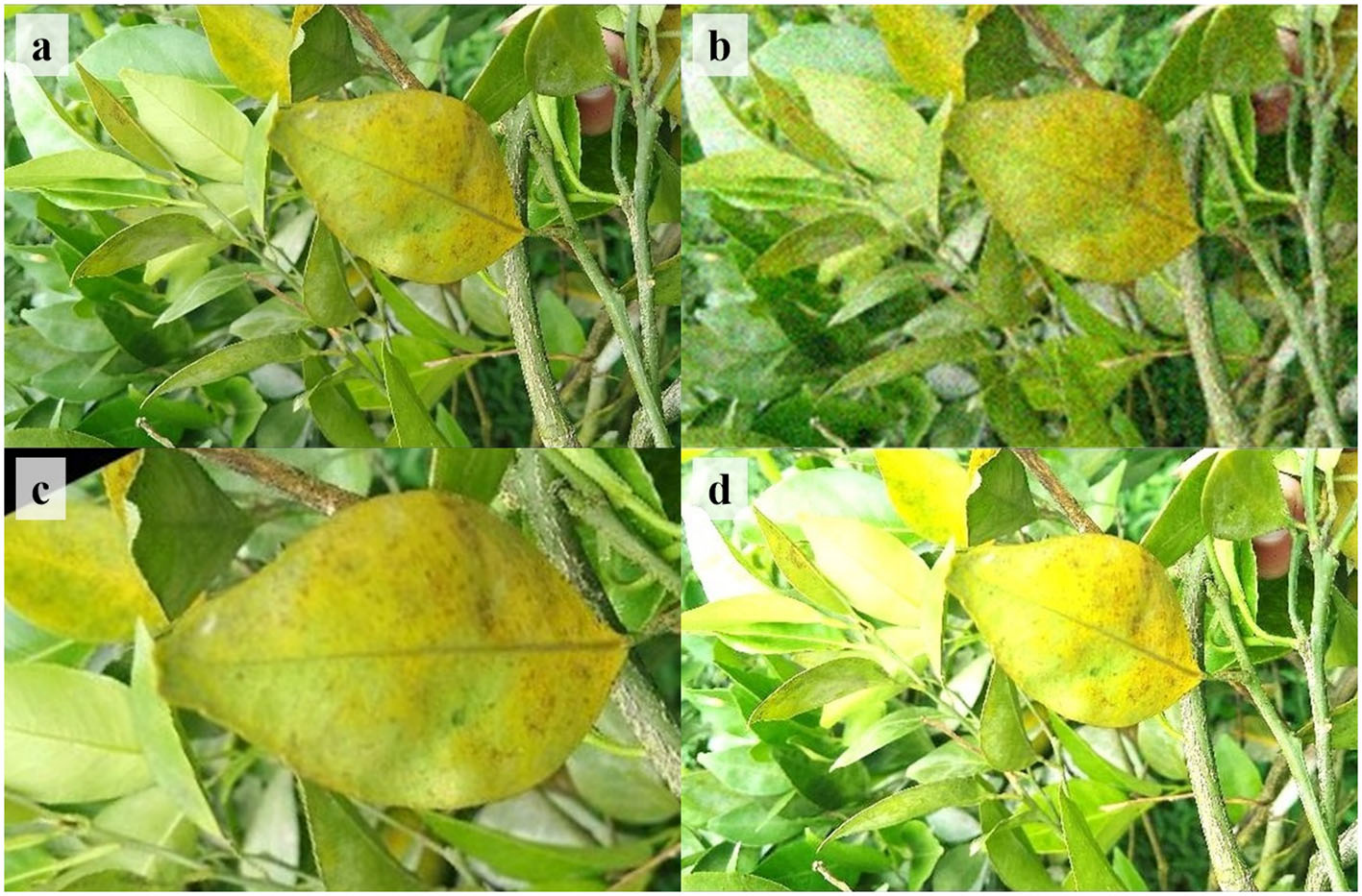
Examples of citrus leaf image augmentation. **(a)** Original image. **(b)** Noise perturbation. **(c)** Rotation. **(d)** Brightness transformation.

Because the initial dataset showed differences in sample size among classes, direct training with this distribution could cause the model to become biased toward classes with larger sample sizes, thereby affecting detection recall and stability for underrepresented classes. To alleviate class imbalance, a differentiated data augmentation strategy was applied to expand samples of different categories and make the class distribution more balanced. The augmentation methods mainly included brightness adjustment, noise perturbation, and random rotation, as shown in **Figure 2**. Brightness adjustment was used to simulate illumination variation caused by canopy shading and direct sunlight, random rotation represented changes in leaf orientation, and noise perturbation approximated sensor and image transmission interference during field acquisition. Detailed statistics of sample numbers for each category before and after data augmentation are summarized in **Table 1**. The original images were first divided into training, validation, and test sets at a ratio of 8:1:1. Data augmentation was then applied only to the training set to avoid overlap between augmented variants of the same original image across different subsets.

**Table 1:** Statistics of sample numbers for each category before and after data augmentation.

Disease	Label	Original (images)	Augmentations	Augmented (new)	Total
Black spot	black_spot	259	B + R + N	777	1036
Citrus greening	greening	360	R + N	720	1080
Citrus canker	canker	591	N	591	1182
Healthy	healthy	357	R + N	714	1071
Greasy spot	greasy_spot	257	B + R + N	771	1028
Algal spot	algal_spot	257	B + R + N	771	1028
Unique images	N/A	2076	N/A	4338	6414

B, brightness adjustment. R, random rotation. N, noise perturbation. The total number represents unique images, while category counts represent annotated instances. Some images contain multiple leaves belonging to different disease categories. Each leaf is annotated using an independent bounding box, and each bounding box is assigned a single class label.

### 2.2 Methods

YOLOv11 is a new generation real time object detection model proposed by Ultralytics. It continues the basic YOLO framework of single stage, end to end, and multi scale prediction, and it uses three detection scales, P3/8, P4/16, and P5/32, to balance fine detail representation for small targets and high level semantic modeling. The detection network consists of a backbone, neck, and head. Features extracted by the backbone are fused at multiple scales and then passed to the detection head for classification and bounding box regression. Because this structure has good real time performance and engineering deployability, YOLOv11 was selected as the baseline framework. For the YOLOv11n model, this study mainly introduced the following structural improvements:

1. In the backbone, the original backbone network was replaced with StarNet-S050. The element wise multiplication mechanism in the Star operation was used to enhance high order nonlinear interactions among channels, thereby improving basic feature extraction in complex backgrounds.
2. In the neck, the key feature fusion unit was modified into C3k2-Star. On the basis of the original cross stage partial connection design, Star based feature transformation was embedded into the multi scale fusion process to enhance coupling between shallow spatial details and deep semantic information.
3. MNS-Head, namely Multi Scale Normalized Shared Detection Head, was proposed. This detection head reduced detection head redundancy through shared convolution, decoupled prediction, and distributional bounding box regression, while improving the consistency of multi scale prediction and bounding box localization accuracy.

Each structural modification was selected to address a specific constraint of citrus disease recognition in natural orchard scenes. StarNet-S050 was used for efficient feature extraction, C3k2-Star for multi scale feature adaptation, and MNS-Head for lightweight shared prediction. Unlike lightweight YOLO variants that optimize the backbone, neck, or detection head as relatively independent components, SKM-YOLOv11 treats these three stages as a coupled design process. The resulting architecture therefore forms a sequential pathway from feature extraction to feature adaptation and prediction rather than a simple collection of replacement modules.

**Figure 3:**
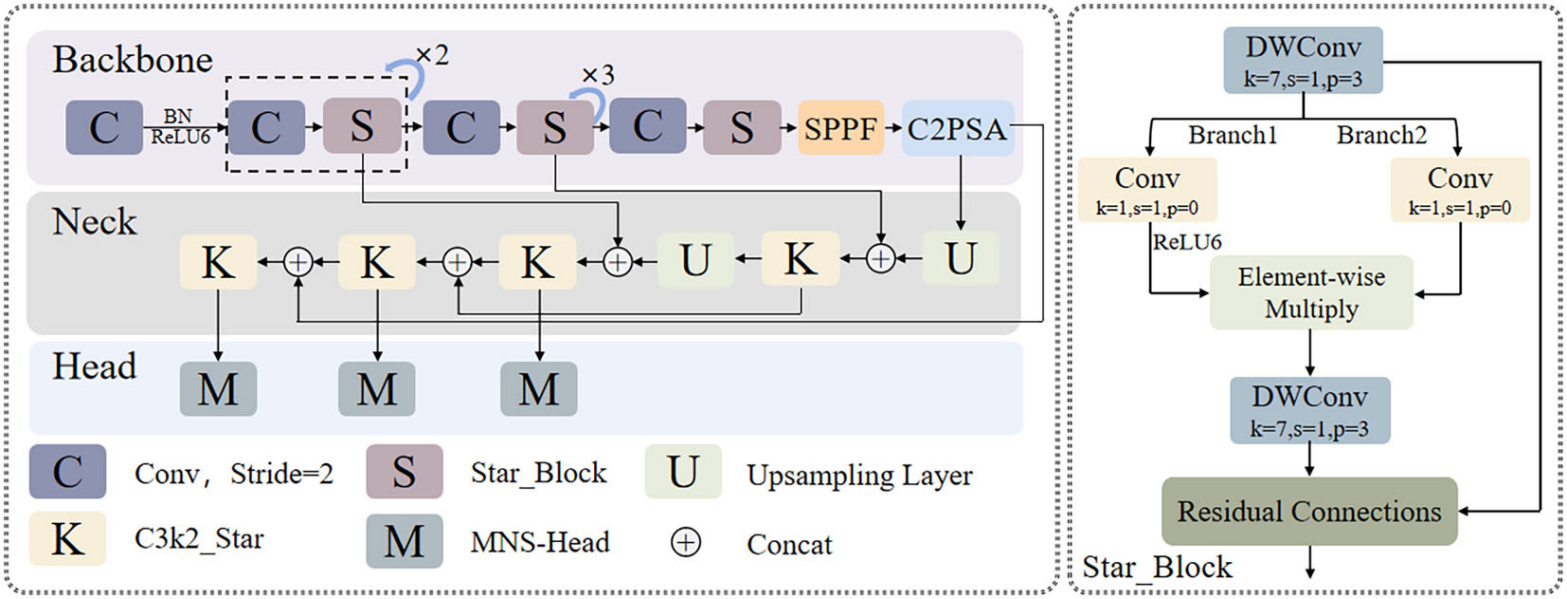
Overall structure of the SKM-YOLOv11 lightweight citrus leaf disease detection model.

The improved model was named SKM-YOLOv11, and its network architecture is shown in **Figure 3**.

#### 2.2.1 Lightweight backbone based on StarNet-S050

**Figure 4:**
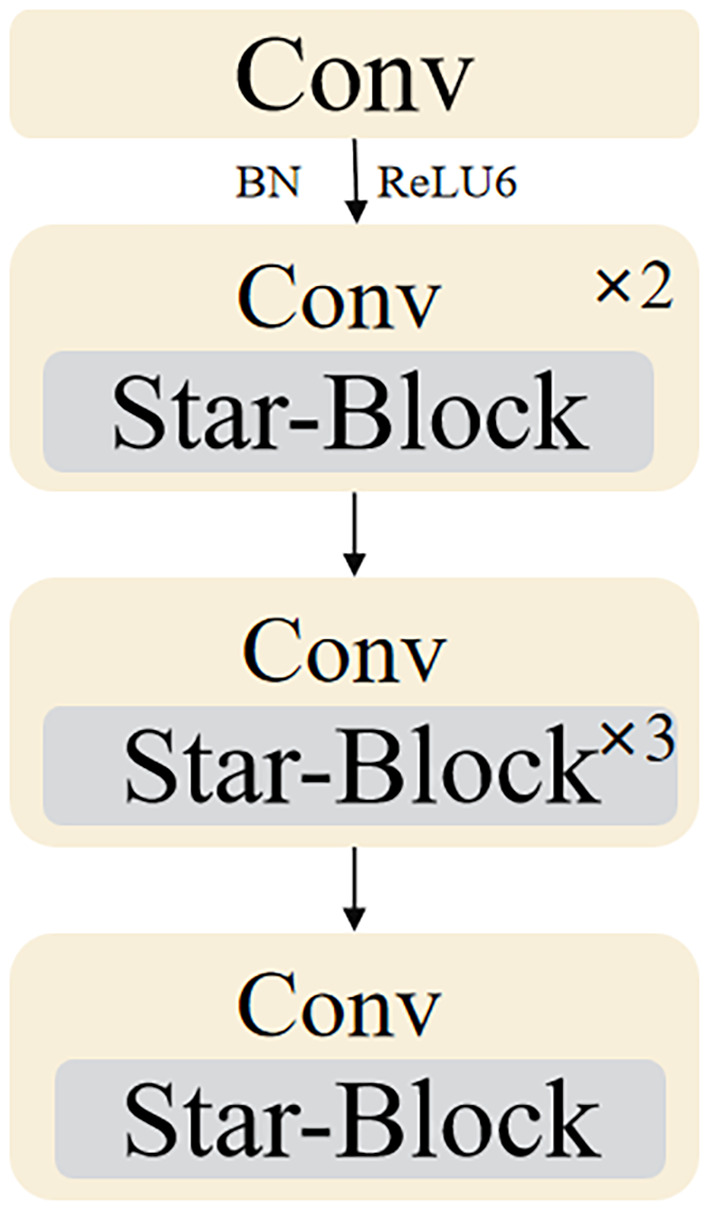
Structure of the StarNet-S050 lightweight backbone network.

Although the original YOLOv11n already has high inference efficiency, its backbone still mainly relies on convolution based local feature extraction units. There remains room for further lightweight optimization and feature representation improvement in complex leaf lesion scenes. Citrus leaf disease symptoms usually show variable scales, subtle textures, blurred boundaries, and strong background interference. Therefore, the network needs strong fine grained feature extraction ability under a relatively low parameter budget (Huang et al., 2024). For this purpose, the compact and efficient StarNet-S050 was introduced as the lightweight backbone network (Ma et al., 2024). The network structure is shown in **Figure 4**.

StarNet-S050 was selected instead of simply reducing the depth or width of YOLOv11n because direct structural scaling may lower computational cost at the expense of fine-grained lesion representation. Unlike architectures that rely on computationally expensive self-attention mechanisms or redundant module stacking, StarNet preserves local spatial filtering through depthwise convolution and introduces multiplicative feature interaction through the Star Operation. This operation provides higher-order nonlinear channel interaction in a low-dimensional feature space without constructing attention matrices (Ma et al., 2024). Such a mechanism is suitable for citrus disease symptoms characterized by weak texture contrast, blurred boundaries, and strong background interference. StarNet-S050 was therefore used to improve the balance between feature representation and computational efficiency at the backbone stage rather than as a generic lightweight replacement.

Specifically, in the basic building block of StarNet, the input features first pass through a depthwise convolution with a kernel size of 7×7 to extract local spatial features and enlarge the local receptive field, thereby strengthening the network’s ability to model lesion textures, lesion boundaries, and local spatial patterns. Subsequently, the features are fed into two parallel 1×1 linear projection layers, followed by nonlinear activation and element wise multiplication. The mathematical formulation of this process is as follows:


$$X_{star}=\sigma (F_{1}(X_{dw}))⊙F_{2}(X_{dw})$$

Where $X_{dw}$ denotes the output of the depthwise separable convolution, $F_{1}$ and $F_{2}$ denote two independent 1×1 convolution layers used to map the channels to an expanded dimension, σ  denotes the ReLU6 activation function, and ⊙ denotes element wise multiplication. This multiplication operation can implicitly introduce high order nonlinear terms without increasing the actual tensor dimension, thereby enhancing the model’s ability to perceive leaf disease boundaries and lesion related texture features.

After the Star Operation, the features are projected back to the original channel dimension through a 1×1 convolution, followed by a second 7×7 depthwise separable convolution, denoted as DWConv, and a DropPath regularization mechanism. The final output is then obtained through a residual connection, which can be expressed as follows:


$$Y=X_{in}+\text{DropPath}({\text{DWConv}}_{2}(G(X_{star})))$$

Through these improvements, the backbone based on StarNet-S050 reduced the overall parameter count, floating point operations, and model size. In addition, its implicit high dimensional feature mapping mechanism reduced interference from redundant background information, enabling the model to focus more effectively and robustly on citrus leaf disease features.

#### 2.2.2 Cross stage feature fusion module based on C3k2-Star

**Figure 5:**
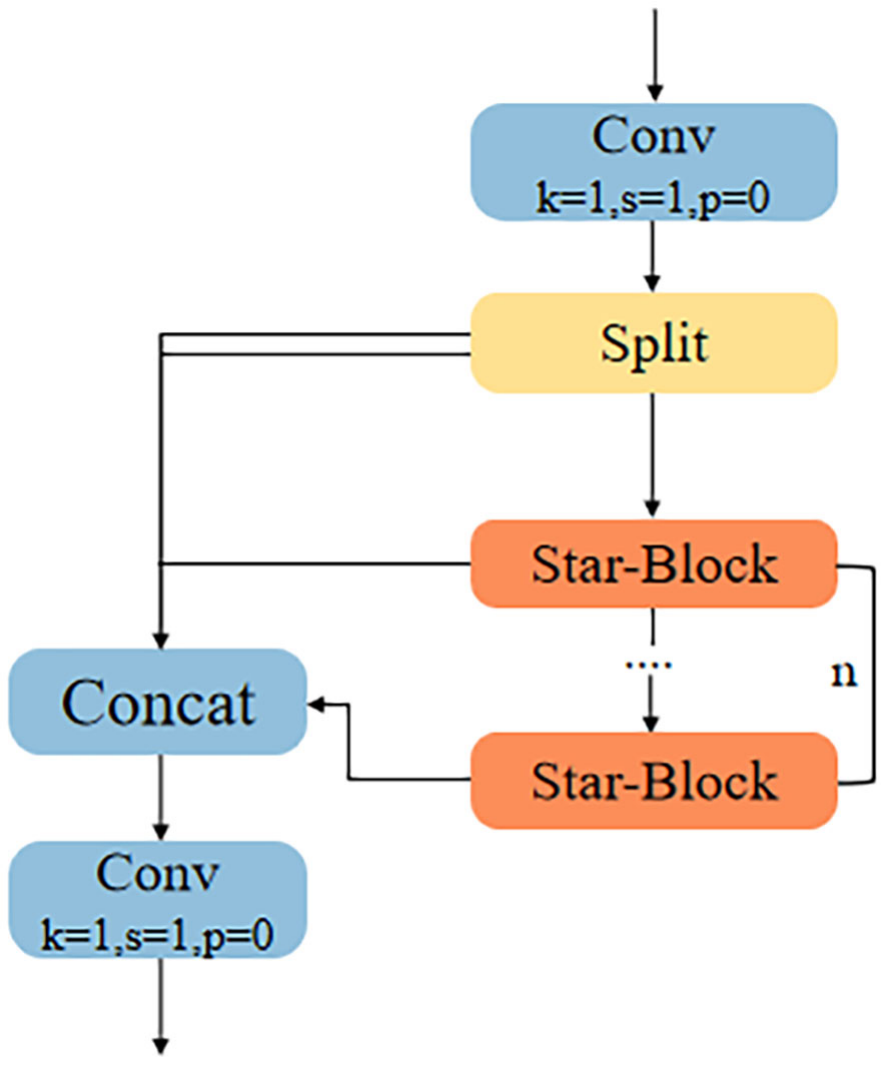
Structure of the C3k2-Star cross stage feature fusion module.

In citrus leaf disease recognition, the neck module of the original YOLOv11n, which is mainly composed of standard C3k2, has certain limitations. On the one hand, repeated stacking of standard convolutional blocks is used internally to enhance feature representation, which introduces a large number of convolutional kernel computations and increases parameter count and computational cost, limiting deployment on agricultural mobile devices. On the other hand, for citrus lesions with variable scales and complex backgrounds, simply increasing the number of convolutional layers is often insufficient to efficiently capture complex feature interactions across scales under limited computing resources. Therefore, the C3k2-Star module was introduced in this study, and its network structure is shown in **Figure 5**.

The role of C3k2-Star was not limited to general feature enhancement. StarNet-S050 substantially compresses the backbone representation and changes the channel composition and statistical characteristics of the multi scale features. In contrast, MNS-Head reuses shared convolutional parameters across different prediction scales and therefore requires relatively consistent feature representations before classification and regression. Directly connecting the lightweight backbone to the shared detection head may result in insufficient channel adaptation and weak semantic alignment across scales. C3k2-Star was therefore introduced as an intermediate adaptation module to recalibrate channel responses, integrate shallow spatial details with deep semantic information, and reduce representation discrepancies among the P3, P4, and P5 features before shared head prediction. This design was intended to mitigate the feature representation gap between StarNet-S050 and MNS-Head.

The core innovation of the C3k2-Star module lies in the integration of the efficient CSPNet macro architecture with the minimal Star Block as the micro computational unit. At the macro level, it inherits the advantages of gradient partitioning and high density feature reuse. The input feature map $X_{in}$ is first subjected to channel adjustment through a 1×1 transition convolution layer $W_{cv1}$,which can be expressed as follows:


$$X_{base}=W_{cv1}*X_{in},$$

where the number of output channels of $W_{cv1}$ is set to twice the hidden channel dimension $c_{h}$ and *denotes the convolution operation. Subsequently, according to the split logic in the code implementation, the resulting feature base is evenly divided into two parts along the channel dimension:


$$\left[X_{1},X_{2}]=\text{Split}(X_{base}\right)$$

where $X_{1}$ is directly transmitted to the end of the module through an identity mapping, preserving part of the shallow features as a shortcut branch to facilitate gradient propagation and enhance feature reuse. In contrast, $\;X_{2}$ is used as the main branch input and is recursively modeled through n successive internal feature transformation units. In the neck configuration adopted in this study, the P3 and P4 branches use the lightweight form with c3k=False,in which the internal unit is the Star_Block. By contrast, the P5 branch uses the form with c3k=True in which the internal unit is C3k-Star, so as to strengthen the representation capability of high level semantic features. The forward propagation process of this branch can be expressed as follows:


$$Y_{0}=X_{2}$$

$$Y_{i}=\text{Star}\_{\text{Block}}_{i}(Y_{i-1}),i\in \{1,2,\ldots ,n\}$$

At the micro computational level, the Star Block was adopted in this study to enhance feature interaction within the hidden channel space. Unlike the conventional C3k2 module, which mainly strengthens representation through parameterized convolutions, the Star series modules improve nonlinear interactions among channels by introducing element wise multiplication, thereby complementing convolutional representation ability with relatively low additional computational cost. For citrus leaf disease detection, this mechanism enables the neck network to capture more complex nonlinear correlations among features at different scales through implicit polynomial mapping without substantially increasing kernel computation. As a result, shallow fine grained lesion boundary features can be fused more effectively with deep global semantic context. The C3k2-Star module collects all intermediate outputs through dense channel concatenation and then maps the concatenated redundant channels back to the output dimension $c_{2}$ through a 1×1transition convolution layer $W_{cv2}$,which can be expressed as follows:


$$Y_{neck\_base}=\text{Concat}(X_{1},Y_{0},Y_{1},\ldots ,Y_{n})$$

$$Y_{out}=W_{cv2}*Y_{neck\_base}$$

This improvement enhances the model representation of variable and subtle agricultural visual features and improves its perception of small scale lesions and marginal diseased regions. At the same time, the module is suitable for deployment on computationally constrained platforms such as embedded orchard monitoring terminals or mobile handheld diagnostic devices. It therefore helps meet the requirements of real time citrus leaf disease detection while maintaining stable mAP@0.5 performance under controlled complexity.

#### 2.2.3 Lightweight shared convolutional detection head MNS-Head (multi scale normalized shared detection head)

**Figure 6:**
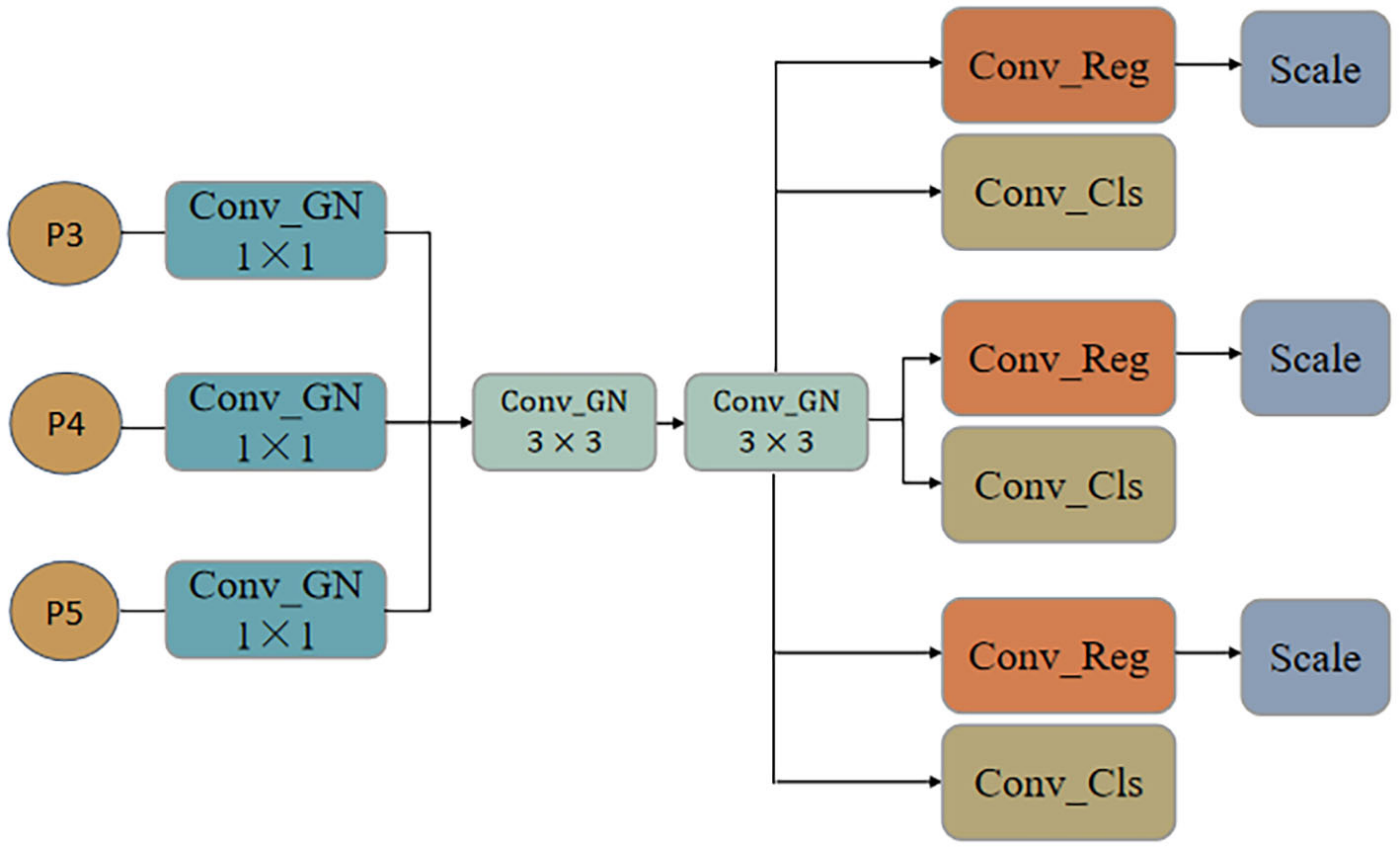
Structure of the MNS-Head lightweight shared convolutional detection head.

In object detection networks, the detection head is responsible for final bounding box regression and category prediction. YOLO series models usually use independent decoupled heads at different feature scales, which causes the detection head to account for a relatively high proportion of the total model parameters. In citrus leaf disease recognition, target regions may vary substantially from small early symptom regions to larger diseased texture regions. To achieve lightweight deployment on edge devices while alleviating semantic differences among features at different scales, this study designed and introduced a Multi Scale Normalized Shared Detection Head, namely MNS-Head, as shown in **Figure 6**.

Independent prediction heads at P3, P4, and P5 repeat similar convolutional transformations and increase parameter overhead. To reduce this redundancy while accommodating differences among multiscale features, MNS-Head first applies an independent 3×3convolutional module to each feature scale for channel alignment and local feature preprocessing. For the multiscale feature maps output by the neck, denoted as $X^{\left(j\right)}$, where j∈{3,4,5}, this process can be expressed as follows:Independent prediction heads at P3, P4, and P5 repeat similar convolutional transformations and increase parameter overhead. To reduce this redundancy while accommodating the differences among multiscale features, MNS-Head first applies an independent convolutional module to each feature scale for channel alignment and local feature preprocessing. For the multiscale feature maps output by the neck, denoted as $X_{i}$, this process can be expressed as follows:


$$F_{adapt}^{\left(j\right)}=\text{Conv}\_{\text{GN}}_{3\times 3}^{\left(j\right)}(X^{\left(j\right)})$$

The aligned features are then processed by a cross-scale shared convolutional block consisting of a shared 3×3depthwise convolution followed by a shared 1×1pointwise convolution. Both convolutional layers are followed by Group Normalization and SiLU activation. The shared transformation can be expressed as follows:


$$F_{shared}^{\left(j\right)}=\text{Shared}\_{\text{Conv}}_{1\times 1}(\text{Shared}\_{\text{DWConv}}_{3\times 3}(F_{adapt}^{\left(j\right)}))$$

This cross-scale parameter-sharing strategy reduces parameter redundancy across the P3, P4, and P5 prediction branches, while the depthwise and pointwise convolutional design further lowers computational cost. However, because multiscale features differ in spatial resolution, receptive field, and statistical distribution, direct weight sharing may increase optimization difficulty or cause performance degradation. Group Normalization was therefore used to stabilize feature normalization without relying on batch-level statistics. In the present implementation, the number of groups was fixed at G=16, and the aligned channel dimension was set to 256, resulting in 16 channels within each normalization group.

In addition, to compensate for the reduced ability of shared regression weights to perceive scale specific information, a learnable scaling factor $S_{\text{j}}$ was introduced into the regression branch, which can be expressed as follows:


$$P_{box}^{\left(j\right)}=S_{j}(W_{reg}*F_{shared}^{\left(j\right)})$$

$$P_{cls}^{\left(j\right)}=W_{cls}*F_{shared}^{\left(j\right)}$$

where $W_{cls}$ and $W_{reg}$ denote the shared 1×1convolution layers used for classification and regression prediction, respectively, and * denotes the convolution operation. Combined with distribution focal loss, MNS-Head is able to maintain high precision localization and classification performance for multi scale citrus disease targets under a low parameter budget, thereby meeting the requirements of real time detection on agricultural mobile devices.

## 3 Results

### 3.1 Experimental platform

The experiments were conducted using the PyTorch deep learning framework under the Windows 11 operating system. The hardware configuration included dual Intel (R) Xeon (R) Silver 4314 processors at 2.40 GHz, an NVIDIA RTX A6000 graphics card, and 512 GB memory. The software environment included Python 3.11.13, PyTorch 2.5.0, CUDA 11.8, CUDA 13.0 system environment, NVIDIA driver 581.15, and PyCharm 2025.1.1.1.

During model training, the maximum number of epochs was set to 300, the batch size was set to 32, and the input image resolution was set to 640 × 640 pixels. Stochastic gradient descent, namely SGD, was used as the optimizer for model training.

### 3.2 Evaluation metrics

Because the proposed model was designed for citrus leaf disease recognition and subsequent deployment on edge devices or resource constrained platforms, it was evaluated from two perspectives: detection performance and deployment efficiency. Precision, Recall, precision recall curves, mAP@0.5, mAP@0.75, and mAP@0.5:0.95 were used to evaluate detection performance. FLOPs, parameter count, and model file size were used to evaluate model complexity and deployment efficiency (Lin et al., 2024; Zhang et al., 2025).

Parameter count was used to measure model scale, FLOPs were used to represent the theoretical computational complexity of a single forward pass, and model file size reflected the actual storage overhead of model weights. In general, lower parameter count and computational cost make a model more suitable for deployment on embedded devices and mobile platforms. A smaller model file size also facilitates model loading, storage, and transmission (Lin et al., 2024; Zhang et al., 2025). Model file size is usually related to parameter count and storage precision and can be approximated as:


$$Size\approx \frac{Params\times b}{8}$$

For detection performance, the PASCAL Visual Object Classes, namely PASCAL VOC, evaluation protocol uses precision recall analysis and mAP as standard object detection evaluation methods and has broad international recognition (Everingham et al., 2015). Precision and Recall are defined as follows:


$$P=\frac{TP}{TP+FP}$$

$$R=\frac{TP}{TP+FN}$$

where TP, FP, and FN denote true positives, false positives, and false negatives, respectively.

mAP@0.5 emphasizes target detection ability, mAP@0.75 requires higher bounding box localization accuracy, and mAP@0.5:0.95 averages average precision across multiple intersection over union, namely IoU, thresholds, thereby providing a more comprehensive measure of overall detection performance (Everingham et al., 2015; Padilla et al., 2021).

For images containing leaves from multiple disease categories, evaluation was performed at the individual bounding box level rather than the image level. Predicted boxes were matched with ground truth boxes according to their class labels and intersection over union values. Each ground truth box could be matched to only one prediction under the corresponding evaluation threshold. Correctly matched predictions were counted as true positives, unmatched predictions were counted as false positives, and unmatched ground truth boxes were counted as false negatives. Therefore, the presence of multiple disease classes in the same image did not result in duplicated metric calculation or conflicting ground truth assignments.

In summary, the improved model was comprehensively evaluated in terms of parameter scale, computational complexity, storage cost, and detection accuracy, thereby reflecting its advantages in the accuracy complexity trade off.

### 3.3 Model training results and analysis

#### 3.3.1 Ablation experiments

To systematically evaluate the effects of each improved module on model lightweight performance and detection performance, YOLOv11n was used as the baseline. StarNet, C3k2-Star, and MNS-Head were individually replaced and combined in different configurations for ablation experiments. The results are shown in **Table 2**.

**Table 2:** Ablation results of SKM-YOLOv11.

S	C	M	P (%)	R (%)	mAP@0.5 (%)	FLOPs (G)	Params	Size (MB)
×	×	×	92.18	92.95	96.15	6.3	2,583,322	5.5
√	×	×	$94.81{↑}^{2.63}$	$93.76{↑}^{0.81}$	$97.44{↑}^{1.29}$	$5.0{↓}^{20.6\%}$	$1,943,538{↓}^{24.8\%}$	$4.0{↓}^{27.3\%}$
×	√	×	$94.13{↑}^{1.95}$	$94.02{↑}^{1.07}$	$97.06{↑}^{0.91}$	$6.4{↑}^{1.6\%}$	$2,472,978{↓}^{4.3\%}$	$5.1{↓}^{7.27\%}$
×	×	√	$95.30{↑}^{3.12}$	$95.43{↑}^{2.48}$	$98.35{↑}^{2.20}$	$5.6{↓}^{11.1\%}$	$2,420,817{↓}^{6.3\%}$	$4.9{↓}^{10.9\%}$
√	√	×	$91.95{↓}^{0.23}$	$94.23{↑}^{1.28}$	$95.99{↓}^{0.16}$	$5.0{↓}^{20.6\%}$	$1,900,130{↓}^{26.4\%}$	$3.9{↓}^{29.1\%}$
√	×	√	$89.95{↓}^{2.23}$	$84.87{↓}^{8.08}$	$91.92{↓}^{4.23}$	$3.5{↓}^{44.4\%}$	$876.537{↓}^{66.1\%}$	$1.9{↓}^{65.5\%}$
×	√	√	$94.51{↑}^{2.33}$	$93.56{↑}^{0.61}$	$96.98{↑}^{0.83}$	$5.7{↓}^{9.5\%}$	$2,310,473{↓}^{10.6\%}$	$4.7{↓}^{14.6\%}$
√	√	√	$95.10{↑}^{2.92}$	$94.42{↑}^{1.47}$	$97.10{↑}^{0.95}$	$4.3{↓}^{31.7\%}$	$1,737,625{↓}^{32.7\%}$	$3.6{↓}^{34.6\%}$

S denotes StarNet-S050, C denotes C3k2-Star, and M denotes MNS-Head. P denotes Precision and R denotes Recall.

The single module results showed clear differences in the contribution of each module to model complexity reduction. Among them, StarNet provided the most significant lightweight improvement. After introducing StarNet, the parameter count decreased from 2.58 M to 1.94 M, FLOPs decreased from 6.3 G to 5.0 G, and model file size decreased from 5.5 MB to 4.0 MB. This indicates that StarNet effectively reduces parameter redundancy and computational cost at the backbone level and is a key component for model compression. Despite the substantial reduction in complexity, Precision, Recall, and mAP@0.5 increased to 94.81%, 93.76%, and 97.44%, respectively, indicating that this module did not weaken detection ability while reducing model size.

By contrast, the main role of C3k2-Star was not to greatly compress model size, but to improve feature representation quality at a relatively low complexity cost by optimizing feature fusion in the neck. Therefore, it mainly provided performance compensation for the lightweight structure. MNS-Head reduced redundancy in the detection branches through shared detection head parameters. It reduced model complexity to a certain extent while providing a clear accuracy gain, indicating a good balance between compression of detection head cost and maintenance of detection performance.

The two module combinations showed different trends. The combination of StarNet and C3k2-Star performed well in lightweight design, further reducing the parameter count to 1.90 M while maintaining an mAP@0.5 of 95.99%. This indicates that the combination can maintain good detection performance at a relatively small model scale. The combination of C3k2-Star and MNS-Head tended more toward performance enhancement, with a more pronounced accuracy improvement but not the best complexity reduction.

Notably, the combination of StarNet-S050 and MNS-Head indicated a feature representation gap between the lightweight backbone and the shared detection head. When StarNet-S050 was directly connected to MNS-Head without C3k2-Star, Precision, Recall, and mAP@0.5 decreased to 89.95%, 84.87%, and 91.92%, respectively, all of which were lower than those of the original YOLOv11n. This degradation was unlikely to result solely from the increased degree of model compression. StarNet-S050 generated highly compact multi scale representations, whereas MNS-Head shared convolutional parameters across prediction scales and therefore required adequately aligned channel semantics and feature statistics. Without C3k2-Star, the direct connection between these two modules provided insufficient feature recalibration and semantic adaptation across scales. After C3k2-Star was incorporated, Precision, Recall, and mAP@0.5 increased to 95.10%, 94.42%, and 97.10%, respectively. These results suggest that C3k2-Star serves as an important adaptation bridge between StarNet-S050 and MNS-Head rather than merely functioning as an optional feature enhancement module.

**Figure 7:**
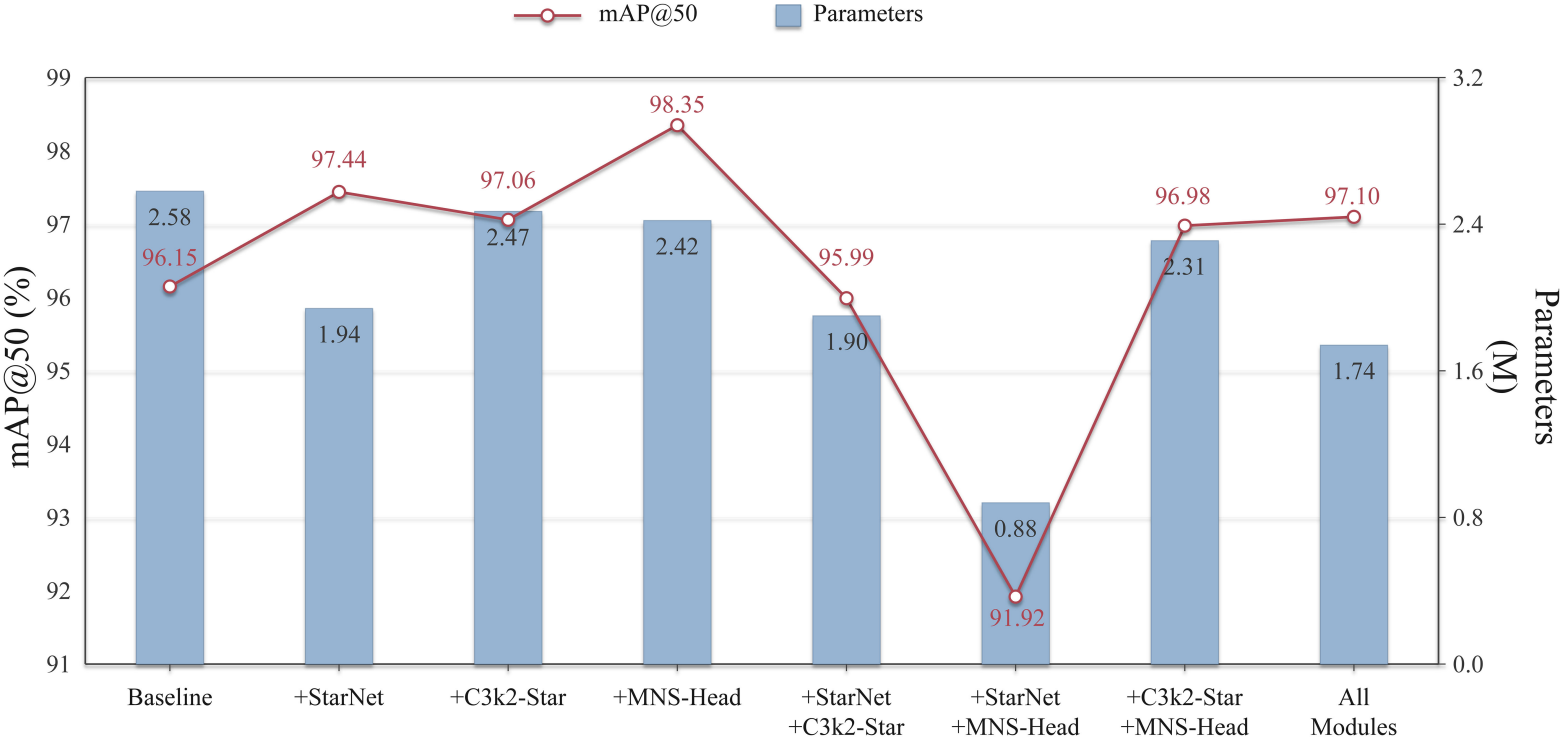
Dual axis comparison of mAP@0.5 and parameter count under different ablation configurations.

As shown in **Figure 7**, the changes in mAP@0.5 and parameter count under different module configurations more intuitively reflect the differences in the roles of the improved modules in accuracy improvement and structural compression.

The ablation results support the proposed problem-driven design logic. The reduction in FLOPs and parameter count after introducing StarNet-S050 confirms its role in backbone compression. The degradation observed when StarNet-S050 was directly combined with MNS-Head confirms that backbone compression and cross-scale head sharing are not automatically compatible. The performance recovery obtained after introducing C3k2-Star supports its role as a multi scale adaptation bridge. The final performance of SKM-YOLOv11 therefore results from coordinated module interaction rather than independent module accumulation.

With the joint action of the three modules, the final SKM-YOLOv11 achieved the best overall performance. Compared with the baseline YOLOv11n, its FLOPs, parameter count, and model file size decreased to 4.3 G, 1.74 M, and 3.6 MB, corresponding to reductions of 31.7%, 32.7%, and 34.55%, respectively. This indicates that the model achieved substantial compression in computational complexity, parameter scale, and storage cost. Meanwhile, Precision, Recall, and mAP@0.5 increased by 2.92, 1.47, and 0.95 percentage points, respectively. These results show that the proposed method achieved lightweight design without sacrificing detection performance and even improved accuracy on the basis of a markedly smaller model scale. In summary, SKM-YOLOv11 achieved a favorable balance between lightweight design and detection performance, making it more suitable for edge deployment in citrus leaf disease recognition. Although the MNS-Head only variant achieved the highest mAP@0.5, it retained higher FLOPs, parameter count, and model size than SKM-YOLOv11. Therefore, the final model was selected based on the overall balance between accuracy and deployment efficiency rather than a single accuracy metric.

#### 3.3.2 Comparative experiments

To further evaluate the overall performance of SKM-YOLOv11, typical object detection models including YOLOv5n, YOLOv6n, YOLOv8n, YOLOv10n, YOLOv11n, YOLOv12n, and YOLO26n were selected for comparative experiments. The evaluation metrics included Precision, Recall, mAP@0.5, mAP@0.75, mAP@0.5:0.95, FLOPs, parameter count, and model file size. The results are shown in **Table 3**.

**Table 3:** Performance and complexity comparison of different detection models.

Model	P (%)	R (%)	mAP@0.5 (%)	mAP@0.75 (%)	mAP@0.5:0.95 (%)	FLOPs (G)	Params (M)	Size (MB)
YOLOv5n	90.33	91.55	93.33	76.43	70.40	7.1	2.50	5.1
YOLOv6n	91.64	89.56	93.64	77.31	69.04	11.7	4.23	8.3
YOLOv8n	90.63	93.46	94.94	80.94	73.41	8.1	3.01	6.0
YOLOv10n	92.94	92.12	94.97	81.90	73.54	6.5	2.27	5.8
YOLOv11n	92.18	92.95	96.15	83.94	74.58	6.3	2.58	5.5
YOLOv12n	91.26	92.63	93.92	76.73	70.81	6.3	2.56	5.6
YOLO26n	94.93	91.04	96.38	79.18	73.13	5.2	2.38	5.2
SKM-YOLOv11	95.10	94.42	97.10	88.96	79.56	4.3	1.74	3.6

SKM-YOLOv11 showed strong overall competitiveness among the compared models. Its Precision, Recall, mAP@0.5, mAP@0.75, and mAP@0.5:0.95 reached 95.10%, 94.42%, 97.10%, 88.96%, and 79.56%, respectively. In terms of accuracy metrics, SKM-YOLOv11 outperformed all other compared models on the main detection metrics, indicating that it has good overall performance in target recognition and localization accuracy. In particular, SKM-YOLOv11 showed more pronounced advantages over several lightweight models in mAP@0.75 and mAP@0.5:0.95, suggesting that it maintained high bounding box localization quality under stricter IoU thresholds.

In terms of model complexity, the FLOPs, parameter count, and model file size of SKM-YOLOv11 were only 4.3 G, 1.74 M, and 3.6 MB, respectively, which were the lowest among all compared models. This indicates that SKM-YOLOv11 had the strongest compression capability in computational cost, model scale, and storage cost. Compared with YOLOv11n, SKM-YOLOv11 achieved accuracy improvement while further reducing complexity. Compared with lightweight models such as YOLOv10n, YOLOv12n, and YOLO26n, SKM-YOLOv11 also showed better overall metrics. These results indicate that model compression was not achieved at the expense of detection performance, but rather that more obvious lightweight benefits were achieved while maintaining high detection accuracy.

**Figure 8:**
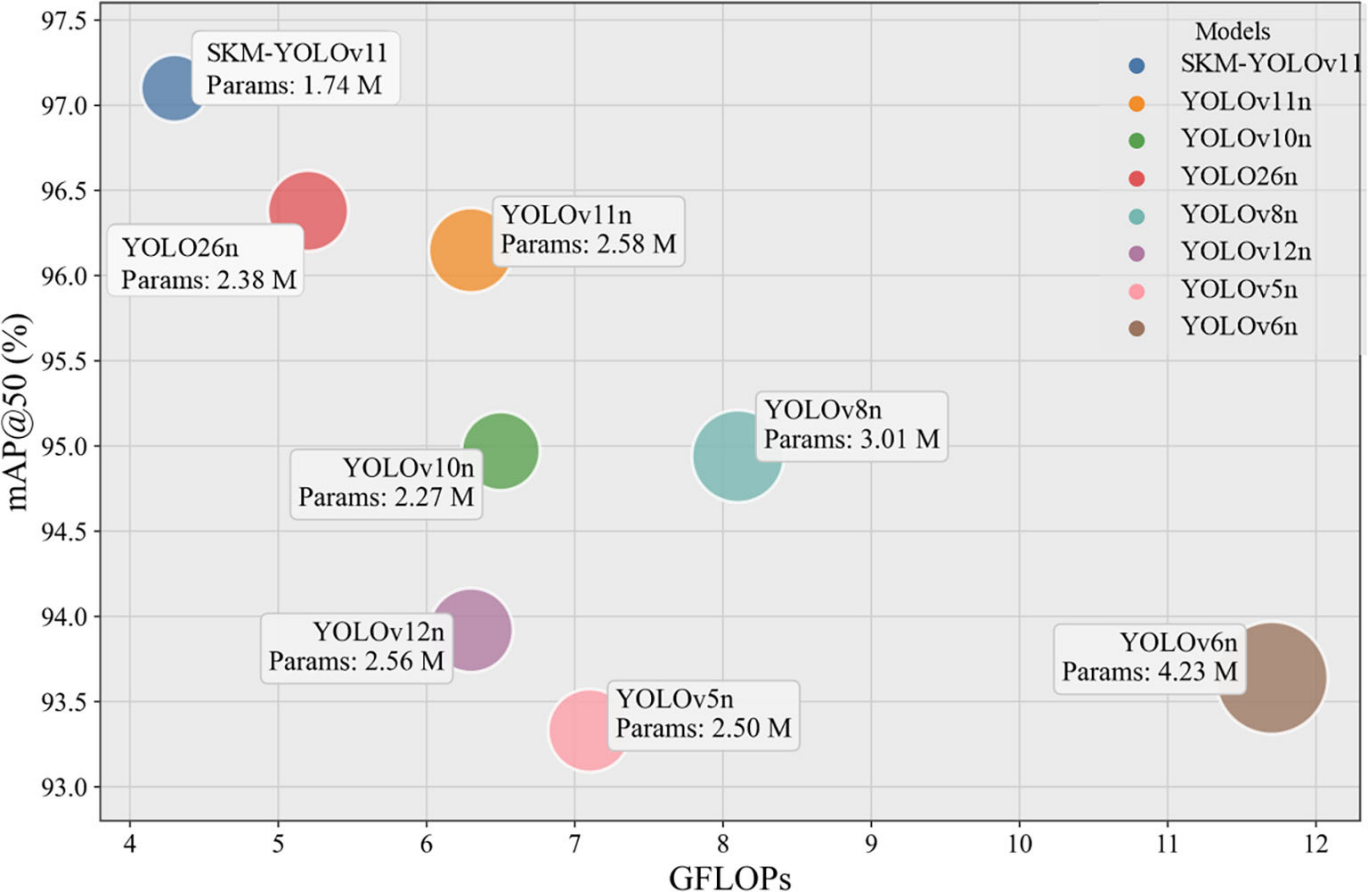
Bubble plot showing the relationship between mAP@0.5 and FLOPs for different object detection models.

The two dimensional bubble plot of FLOPs and mAP@0.5 shown in **Figure 8** further reflects the tradeoff among different models. SKM-YOLOv11 is located closer to the upper left region of the plot, indicating that it achieved higher mAP@0.5 under lower FLOPs and therefore had a better balance between accuracy and complexity.

**Figure 9:**
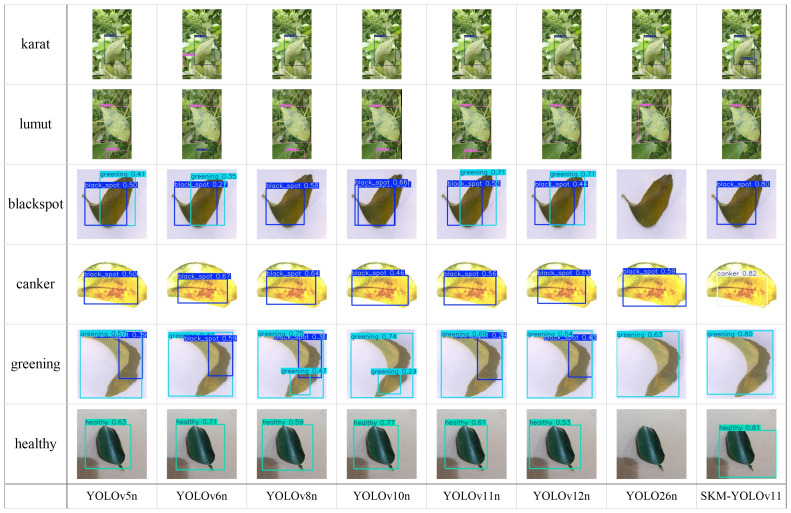
Comparison of detection results of different models on various citrus leaf disease samples.

To visually verify the detection performance of SKM-YOLOv11, **Figure 9** shows the visualization results of different models on samples of various citrus leaf diseases. Because different disease classes show strong similarities in color, texture, and morphology, the recognition task is difficult and may easily lead to false detections, missed detections, and localization deviations. As shown in **Figure 9**, clear differences were observed in the detection performance of the compared models under complex backgrounds and fine grained lesion scenes. Except for SKM-YOLOv11, the other models showed missed detections, false detections, or inaccurate localization to varying degrees. In contrast, the proposed SKM-YOLOv11 showed better detection accuracy and stability under the same interference conditions. It could more accurately recognize different disease classes and effectively reduce false detections and missed detections, indicating stronger feature representation and target discrimination ability in complex scenes.

### 3.4 Edge device deployment

**Figure 10:**
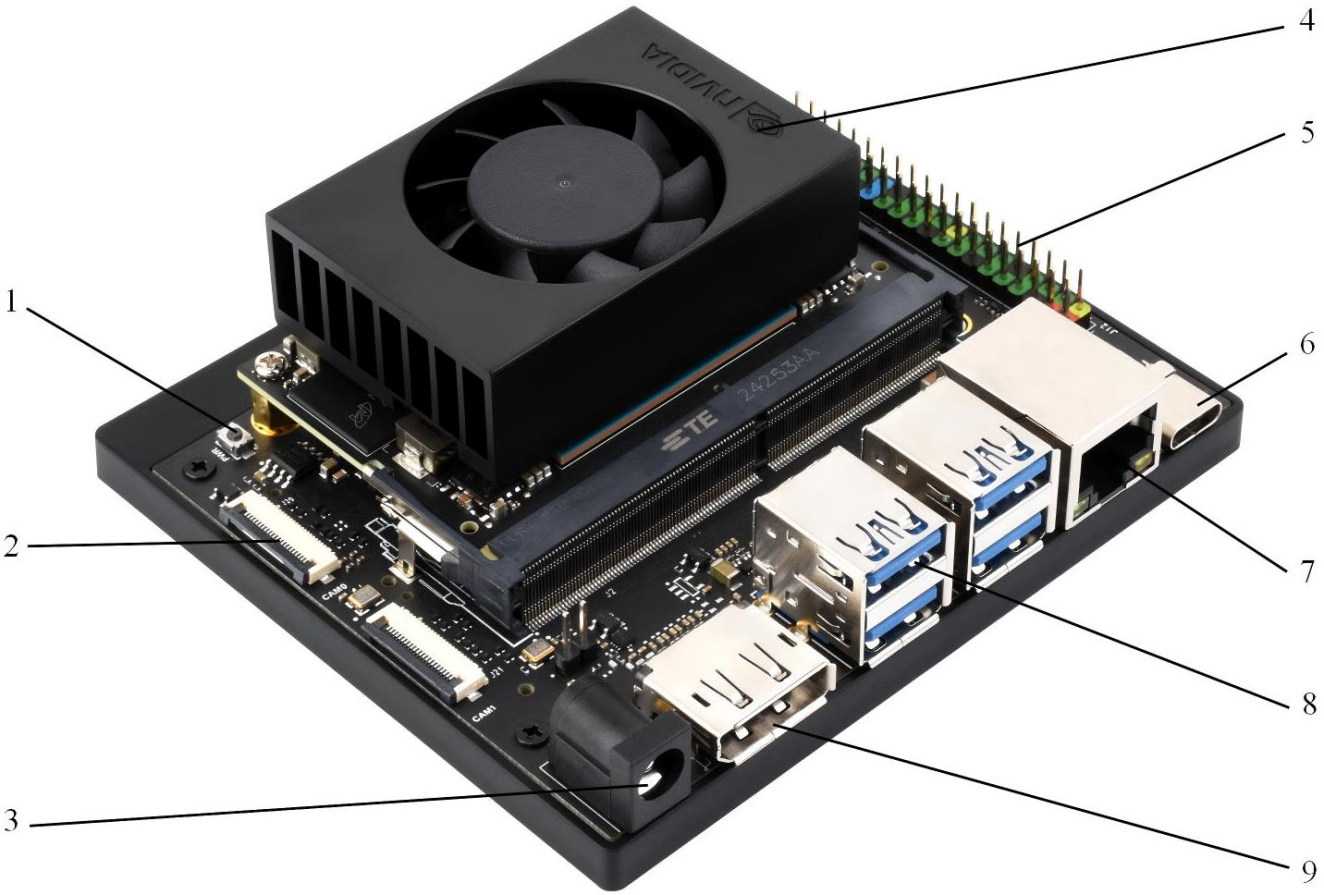
Edge deployment platform of SKM-YOLOv11 on Jetson Orin NX Super. 1. Power button. 2. CSI interface. 3. Power interface. 4. Heat sink. 5. 40 pin GPIO interface. 6. Type-C interface. 7. Ethernet interface. 8. USB interface. 9. DP interface.

To further verify the feasibility of deploying the proposed SKM-YOLOv11 model on edge devices, the model was deployed on an NVIDIA Jetson Orin NX 16 GB edge computing platform, as shown in **Figure 10**. The platform integrates an NVIDIA Ampere architecture GPU with Tensor Cores and an Arm CPU, providing sufficient computing capability for low power real time inference. The deployment environment included JetPack 6.2, CUDA 12.6, cuDNN 9.3, TensorRT 10.3, and Python 3.10.12. The trained PyTorch model was exported to ONNX format using opset 17 with a fixed input tensor size of 1 × 3 × 640 × 640. The ONNX graph was simplified using onnxsim 0.4.36 and subsequently converted into a TensorRT inference engine using trtexec. The engine was built with FP16 precision and a batch size of 1. During testing, the Jetson Orin NX operated in MAXN SUPER mode, and the CPU and GPU frequencies were locked at their maximum values using jetson_clocks. The TensorRT conversion settings, input resolution, batch size, and inference precision were kept unchanged throughout all deployment experiments to ensure a consistent and fair evaluation.

In the deployment test, test images that were not used during training were randomly selected and deployed on Jetson Orin NX Super. SKM-YOLOv11 achieved Precision, Recall, and mAP@0.5 values of 95.60%, 93.17%, and 97.02%, respectively, indicating that the improved model maintained high detection accuracy on the edge device. The deployment test also showed that the model reached a detection speed of 72.45 frames per second. Overall, the improved model balanced detection accuracy and real time inference capability under edge deployment conditions, indicating practical potential for edge intelligent detection in agricultural scenarios such as citrus leaf disease recognition.

## 4 Discussion

### 4.1 Accuracy and lightweight balance mechanism of SKM-YOLOv11 and its adaptability to complex natural scenes

SKM-YOLOv11 achieved lightweight design while maintaining detection performance mainly through coordinated optimization among the backbone, feature fusion module, and detection head, rather than through a single module. StarNet-S050 reduced redundant computation at the backbone level and improved basic feature extraction efficiency. C3k2-Star strengthened fusion across scales between shallow details and deep semantics, compensating for possible feature representation limitations under lightweight conditions. MNS-Head reduced parameter redundancy in the detection head through shared convolution while maintaining classification and localization ability. Together, these three components allowed the model to focus on key features related to disease recognition under limited computing resources, thereby achieving a balance between accuracy and lightweight design.

The marked degradation observed in the configuration combining StarNet-S050 and MNS-Head further clarifies the cooperative mechanism of the proposed architecture. Backbone compression and detection head sharing impose simultaneous constraints on feature richness and interscale consistency. When these two strategies are applied without an intermediate adaptation module, the compact backbone outputs cannot provide sufficiently aligned representations for shared head prediction, resulting in a feature representation gap and substantial reductions in Recall and mAP@0.5. C3k2-Star addresses this problem by recalibrating channel responses and coordinating spatial and semantic information across different feature resolutions before prediction. The performance recovery observed in the complete configuration combining StarNet-S050, C3k2-Star, and MNS-Head is therefore consistent with the proposed role of C3k2-Star as the interface that enables effective cooperation between StarNet-S050 and MNS-Head.

Beyond the architectural coordination described above, the performance of the proposed method in complex natural scenes is also related to the visual characteristics of the task and the annotation strategy. Citrus leaves in orchard canopies are frequently affected by mutual occlusion, dense foliage, illumination variation, and background clutter. In addition, disease symptoms may consist of several small and discontinuous lesions distributed across the same leaf. Under these conditions, lesion level annotation would generate numerous small or overlapping targets and increase the burden of prediction and postprocessing on embedded devices. Whole leaf annotation allowed the model to integrate local lesion appearance with leaf contour, vein texture, and symptom distribution while maintaining a relatively small number of detection targets. This design was therefore more consistent with the objective of rapid leaf level disease screening under edge deployment constraints. However, it was not intended for precise lesion segmentation or disease severity estimation.

### 4.2 Lightweight compression boundary, engineering application value, and limitations

The results of this study indicate that stronger lightweight compression is not always better. Instead, there is an appropriate boundary that matches task requirements. For fine grained tasks such as citrus leaf disease recognition, the model needs not only low complexity but also sufficient discriminative ability for weak textures, small lesions, and complex backgrounds. Excessive compression may further reduce parameter count and computational cost, but it can also weaken feature representation ability and affect detection stability. Therefore, the final SKM-YOLOv11 did not simply pursue the smallest model size. Instead, it achieved a favorable balance among detection performance, model complexity, and deployment requirements, which constitutes its practical value.

From an application perspective, the deployment results on Jetson Orin NX Super indicate that SKM-YOLOv11 is suitable not only for offline analysis in experimental environments but also has the potential for real time operation on smart orchard inspection terminals and edge devices. Nevertheless, this study still has several limitations. First, whole leaf annotation is more suitable for disease category recognition and rapid screening, but it provides limited support for lesion level fine localization and severity assessment. Second, the scene coverage of the current dataset remains limited, and the generalization ability of the model across regions, seasons, and acquisition devices still needs further validation. Future work may combine more natural scene data, lesion level fine annotation, and quantization compression methods to further improve practical applicability.

## 5 Conclusion

To address strong background interference, large variation in lesion scale, and limited edge deployment in citrus leaf disease recognition under natural scenes, this study proposed a lightweight object detection model, SKM-YOLOv11, based on YOLOv11n. Through coordinated improvements to the backbone, feature fusion module, and detection head, the model reduced complexity while improving disease recognition performance. It provides a feasible solution for rapid citrus leaf disease detection and edge deployment in complex natural scenes. The main conclusions are as follows.

1. This study constructed the lightweight detection model SKM-YOLOv11. By introducing StarNet-S050, C3k2-Star, and MNS-Head, coordinated optimization was achieved at three levels: backbone feature extraction, multi scale feature fusion, and detection head design. This enhanced the representation of fine grained disease features under complex backgrounds.
2. The experimental results showed that SKM-YOLOv11 significantly reduced model complexity while maintaining detection accuracy. Compared with YOLOv11n, Precision, Recall, and mAP@0.5 increased by 2.92, 1.47, and 0.95 percentage points, respectively. FLOPs, parameter count, and model file size decreased to 4.3 G, 1.74 M, and 3.6 MB, respectively. These results indicate that the proposed method achieved a favorable balance between accuracy and lightweight design.
3. The edge deployment results showed that SKM-YOLOv11 maintained high detection accuracy and good real time performance on the Jetson Orin NX Super platform. This indicates that the model has practical potential in smart orchard inspection, mobile diagnosis, and edge based disease recognition. Future work may further integrate more complex natural scene data, lesion level fine annotation, and model compression methods to improve generalization ability and practical application value.

## Data Availability

The original contributions presented in the study are included in the article/supplementary material. Further inquiries can be directed to the corresponding author.
